# Primary progressive aphasia: a clinical approach

**DOI:** 10.1007/s00415-018-8762-6

**Published:** 2018-02-01

**Authors:** Charles R. Marshall, Chris J. D. Hardy, Anna Volkmer, Lucy L. Russell, Rebecca L. Bond, Phillip D. Fletcher, Camilla N. Clark, Catherine J. Mummery, Jonathan M. Schott, Martin N. Rossor, Nick C. Fox, Sebastian J. Crutch, Jonathan D. Rohrer, Jason D. Warren

**Affiliations:** 1 Department of Neurodegenerative Disease, Dementia Research Centre, UCL Institute of Neurology, Queen Square, London, WC1N 3BG UK; 2 Division of Psychology and Language Sciences, University College London, London, UK

**Keywords:** Primary progressive aphasia, Semantic dementia, Logopenic aphasia, Frontotemporal dementia, Alzheimer’s disease

## Abstract

The primary progressive aphasias are a heterogeneous group of focal ‘language-led’ dementias that pose substantial challenges for diagnosis and management. Here we present a clinical approach to the progressive aphasias, based on our experience of these disorders and directed at non-specialists. We first outline a framework for assessing language, tailored to the common presentations of progressive aphasia. We then consider the defining features of the canonical progressive nonfluent, semantic and logopenic aphasic syndromes, including ‘clinical pearls’ that we have found diagnostically useful and neuroanatomical and other key associations of each syndrome. We review potential diagnostic pitfalls and problematic presentations not well captured by conventional classifications and propose a diagnostic ‘roadmap’. After outlining principles of management, we conclude with a prospect for future progress in these diseases, emphasising generic information processing deficits and novel pathophysiological biomarkers.

**Electronic supplementary material:**

The online version of this article (10.1007/s00415-018-8762-6) contains supplementary material, which is available to authorized users.

## Introduction

The primary progressive aphasias (PPA) are a diverse group of disorders that collectively present with relatively focal degeneration of the brain systems that govern language. Despite much recent attention in the scientific literature [1, 2], these ‘language-led dementias’ remain daunting for even experienced clinicians to diagnose and manage. This is not surprising: PPA is uncommon (estimated prevalence is conservatively around three cases per 100,000 [3, 4]), the underlying pathology is heterogeneous and generally inaccessible and the functions principally targeted are uniquely complex. Although patients with PPA have been described for well over a century [5], the true significance of these disorders was only appreciated quite recently [6, 7] and the paradigm of selective brain network degeneration caused by pathogenic protein spread has transformed our understanding of neurodegenerative disease [8]. While challenging, accurate clinical diagnosis of PPA is worth striving for: these patients are often affected in late middle life, with devastating implications for family life, work and social functioning.

**Fig. 1:**
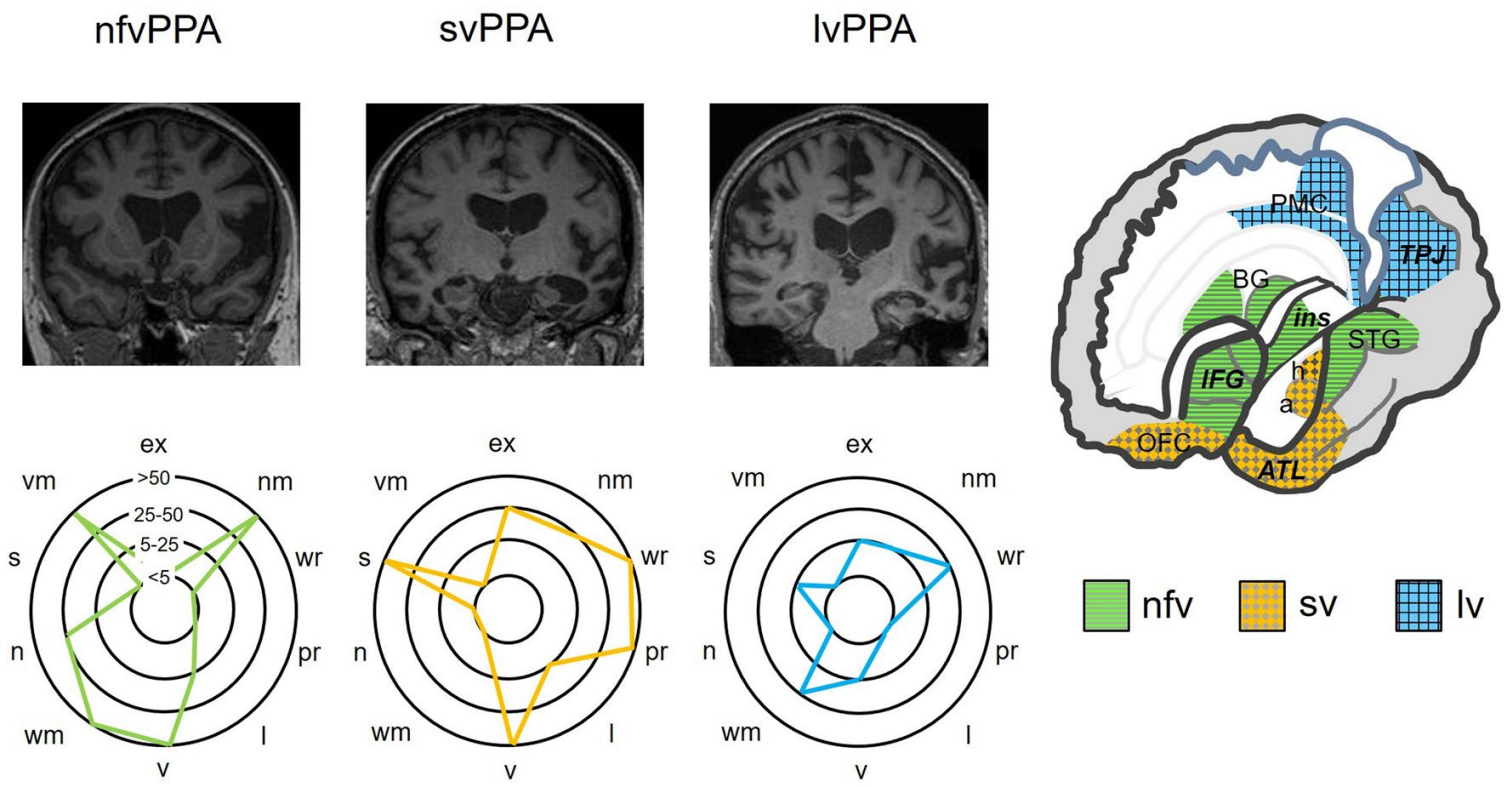
Neuroanatomical and cognitive profiles of the canonical syndromes of progressive aphasia. The top panels present coronal T1-weighted brain MRI sections (in radiological convention, with the left hemisphere on the right) of patients with typical syndromes of nonfluent–agrammatic variant primary progressive aphasia (nfvPPA), showing asymmetric (predominantly left sided) inferior frontal, insular and anterior–superior temporal gyrus atrophy; semantic variant primary progressive aphasia (svPPA), showing asymmetric (predominantly left sided) anterior inferior and mesial temporal lobe atrophy; and logopenic variant primary progressive aphasia (lvPPA), showing atrophy predominantly involving left temporo-parietal junction (posterior–superior temporal and inferior parietal cortices). The cut-away brain schematic (right) indicates the distributed cerebral networks involved in each syndrome; the left cerebral hemisphere is projected forward and major neuroanatomical associations are in bold italics: a, amygdala; ATL, anterior temporal lobe; BG, basal ganglia; h, hippocampus; IFG, inferior frontal gyrus/frontal operculum; ins, insula; OFC, orbitofrontal cortex; PMC, posterior medial cortex (posterior cingulate, precuneus); STG, superior temporal gyrus; TPJ, temporo-parietal junction. The ‘target diagrams’ below show typical profiles of neuropsychological test performance for each syndrome; concentric circles indicate the percentile scores relative to a healthy age-matched population and the distance along the radial dimension represents the level of functioning in the following cognitive domains: ex, executive skills; l, literacy skills; n, naming; nm, nonverbal memory; pr, phrase repetition; s, sentence processing; v, visuo-spatial; vm, verbal memory; wm, word meaning; wr, word repetition

**Fig. 2:**
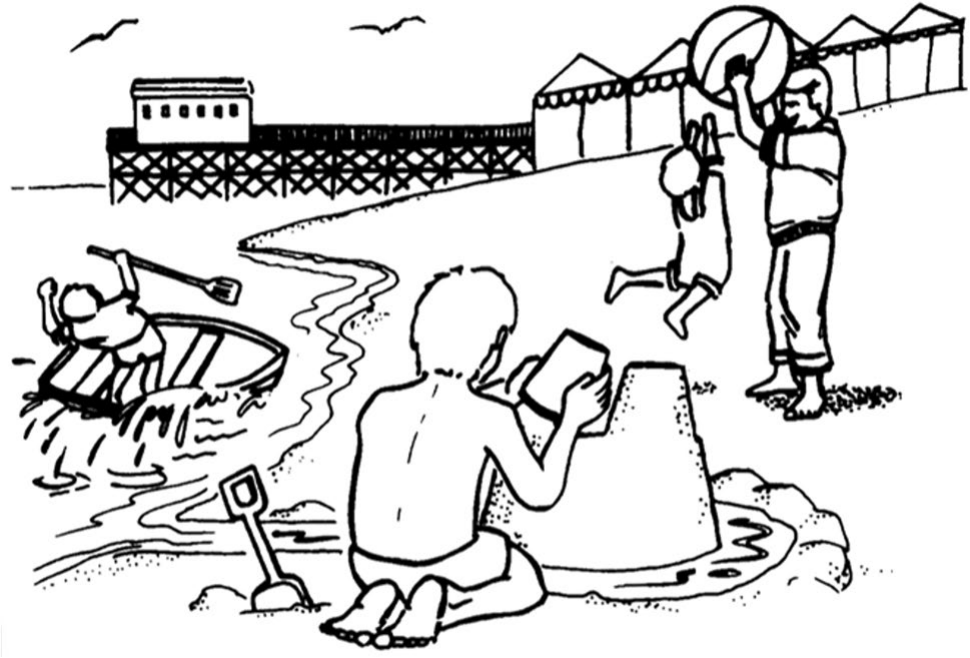
Example of a picture that can be used to elicit conversational speech (reproduced with permission of Professor EK Warrington). A scene of this kind can be used to assess naming and also to probe aspects of language comprehension, at the level of single words (using questions such as, ‘Where is the sandcastle?’) and grammatical relations embodied in sentences (using instructions such as, ‘Point to the thing that the boy is holding above the boat’)

**Fig. 3:**
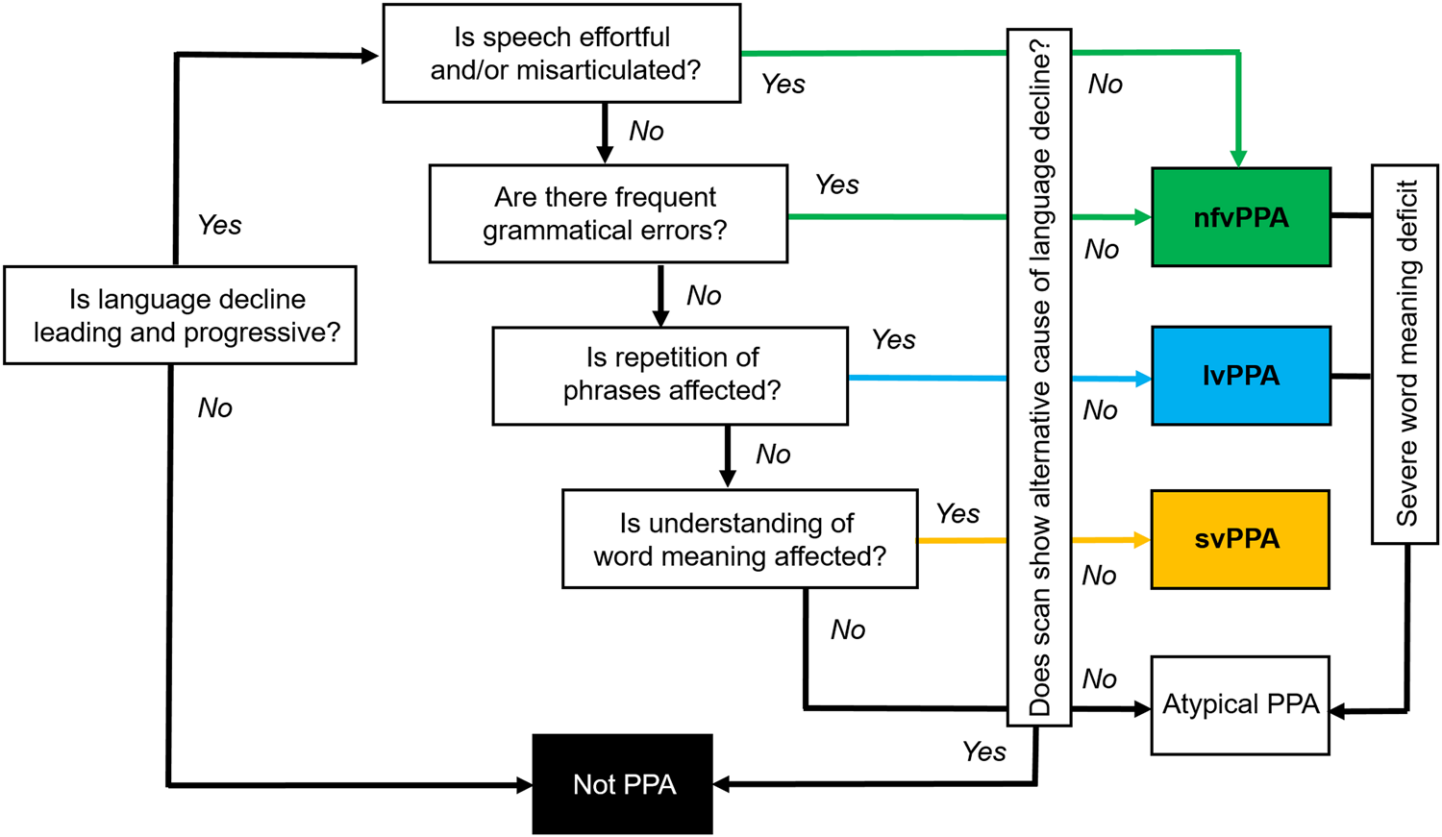
A clinical ‘roadmap’ for diagnosis of canonical primary progressive aphasia syndromes, synthesising key features on history and examination. The ‘forks’ comprising the middle section of the map indicate major decision points, for corroboration using the more detailed framework presented in Table 2. Neuropsychological assessment (where available) is used both to support and quantify the clinical impression and to reveal additional cognitive deficits that may not be emphasised in the clinic but define the overall syndrome (see Fig. 1). Brain imaging (wherever feasible, MRI) is essential to rule out brain tumours and other non-degenerative pathologies that can occasionally present with progressive aphasia; it also has an important ‘positive’ role in corroborating the neuroanatomical diagnosis (see Fig. 1). Ancillary investigations such as CSF examination are used to stratify pathologies within particular syndromes (e.g., lvPPA), with a view to prognosis and treatment. A significant minority of cases will not be diagnosed by this algorithm, falling into the still poorly defined category of ‘atypical’ progressive aphasias (see text and Table 1). lvPPA, logopenic variant primary progressive aphasia; nfvPPA, nonfluent–agrammatic variant primary progressive aphasia; svPPA, semantic variant primary progressive aphasia

In this review, we outline an approach to the diagnosis and management of PPA in the clinic and at the bedside, distilled from our accumulated experience of meeting and caring for these patients. We firstly present a clinical framework for assessing language functions, tailored in particular to the major syndromic presentations of PPA (Tables 1 and 2, Figs. 1 and 2). We then consider these presentations in detail. Three major forms of PPA—nonfluent–agrammatic variant (nfvPPA), semantic variant (svPPA) and logopenic variant (lvPPA)—comprise the canonical syndromes currently recognised in consensus diagnostic criteria [9] (see Table S1, Supplementary Material on-line). These syndromes are distinguished by the language deficits with which they present and associated cognitive, neurological and neuroanatomical profiles and tend to have distinct neuropathological substrates. Key features of PPA syndromes are summarised in Tables 1, 2 and 3 and Fig. 1; additional ‘clinical pearls’ that we have found useful in diagnosis of each syndrome (but which are not widely discussed in the literature of these conditions) are presented in Table 4. Following the taxonomy of classical (stroke) aphasiology, nfvPPA might be anticipated to align with Broca’s aphasia, svPPA with transcortical sensory aphasia and lvPPA with Wernicke’s or conduction aphasia. However, such clinical correspondences are loose, at best. This probably reflects the very different nature of the underlying disease processes, and most pertinently, the distributed neural network basis of PPA [10]. One important corollary is that PPA syndromes extend (cognitively and neuroanatomically) beyond the province of language, to involve other complex behavioural functions. The clinical challenges posed by PPA foreshadow significant unresolved issues in the nosology and neurobiology of these conditions [1, 2, 10–16]. Here we highlight potential diagnostic pitfalls including atypical variant presentations of PPA not well captured by standard criteria (Table 1) and propose a diagnostic ‘roadmap’ (Fig. 3). After outlining principles of management of PPA, we conclude with a prospect for future developments.

**Table 1:** Summary of key language features and cognitive, neurological, neuroanatomical and neuropathological associations in syndromes of primary progressive aphasia

Syndrome	Message production				Message understanding		Speech repetition		Other cognitive and behavioural deficits	Associated neurological	Neuroanatomy	Pathology
	Idea	Content	Structure	Delivery	Perception	Meaning	Words	Phrases				
Canonical												
Nonfluent–agrammatic	±	±	+^a^	+^b^	±	±^c^	+	+	Dysexecutive, orofacial > limb apraxia	Parkinsonism, PSP, CBS; some MND	L ant peri-Sylvian, subcortical	Most often tauopathy (may be PSP, CBD); also AD, TDP-43
Semantic	–	+	–	–	–	+	–	–	Prosopagnosia, visual agnosia, other agnosias; disinhibition, lack of empathy, obsessions	Usually none	L > R ant TL (most marked inferior, mesial)	Usually TDP-43 (type C); some tauopathy, AD, rarely mutations
Logopenic	±	+^d^	+^e^	–	±	±^c^	±	+	Reduced digit span, limb apraxia, acalculia, visuo- spatial agnosia	Myoclonus	L peri-Sylvian, early TPJ	Usually AD
Variant and atypical												
Primary progressive apraxia of speech [20]	–	–	–	+^b^	–	–	+^b^	+^b^	Usually orofacial apraxia, may have dysexecutive, limb apraxia	Parkinsonism, PSP, CBS; rarely MND	Bilat FL-subcortical	Usually tauopathy (may be PSP, CBD)
Mixed progressive aphasia [11]	±	+	+	±	±	+	+	+	Variable—often dysexecutive, parietal	Parkinsonism	L > R peri-Sylvian, ant TL	May have *GRN* mutation, AD, Pick’s
Progressive dynamic aphasia [102]	+^f^	±	±	±	–	–	–	–	Dysexecutive	Parkinsonism, PSP, CBS	Bilat FL-subcortical	May be PSP, CBD
Progressive pure anomia [103]	±	+	–	–	–	–	–	–	None	None	L > R ant TL	Uncertain; ?TDP-43, AD
Progressive dysprosodia [26]	–	–	–	+^g^	±	–	+^g^	+^g^	Dysexecutive, orofacial apraxia	Uncertain	R frontotemporal	Uncertain
Progressive ‘pure’ word deafness [104]	–	–	±	±	+^h^	−^i^	+	+	Cortical deafness, auditory agnosia	Variable	L peri-Sylvian, TPJ	May be unusual, e.g. prion

The Table presents the major (canonical) syndromes of progressive aphasia, as recognised in current consensus diagnostic criteria (see Table S1) and other less common variants and atypical syndromes that are also represented in most clinics seeing patients with progressive aphasia (numbers in brackets designate primary references). Language features refer to functions described in Table 2. **+**, prominent or defining impairment; **±** , variable impairment; −, mild or no deficit (in the majority of cases, problems will first be noted with speech but occasional patients present initially with reading or writing impairments [101])

*AD* Alzheimer’s disease, *ant* anterior, *CBD/S* corticobasal degeneration/syndrome, *FL* frontal lobe, *GRN* progranulin gene, *L* left, *MND* motor neuron disease, *PSP* progressive supranuclear palsy, *R* right, *TDP-43* TAR DNA-binding protein 43, *TL* temporal lobe, *TPJ* temporo-parietal junction

^a^Variable prominence of grammatical and/or speech sound (syllabic) errors

^b^Speech apraxia with prominent speech sound (phonetic, articulatory) errors

^c^Impaired comprehension of more complex sentences (comprehension of single words usually relatively spared)

^d^Typically long word-finding pauses

^e^Speech sound (phonological) errors, grammar usually intact

^f^The patient appears to have ‘nothing to say’ spontaneously but language (if it can be induced by a specific context) is relatively normal in content, structure and delivery and generation of new nonverbal ideas can sometimes be demonstrated in other vocal domains such as singing

^g^Impaired speech rhythm and ‘melody’ or altered accent with infrequent speech sound errors

^h^Difficulty understanding speech disproportionate to any peripheral hearing loss

^i^Much better comprehension of written than spoken messages earlier in the course

**Table 2:** A framework for assessment of language functions, directed particularly to progressive aphasias

Communication task	Cognitive process	Key history	Clinical test	Neuropsychological test	SYND
Message production					
Idea	Generating verbal idea	Reduced initiation of conversation^a^	Describe a recent vacation; generating words by initial letter (e.g. ‘F’) or category (e.g., ‘animals’)^a^	Verbal fluency (F-A-S, category)^d^	DA
Content	Word (vocabulary) retrieval	Unable to find right words (especially names), circumlocutions, pauses	Naming pictures or from verbal description	Graded Naming Test^e^	SV, LV, PA
Structure	Sentence assembly	Grammatical errors (especially in writing or electronic media)	Written sentence production	Argument Structure Production Test^f^	NFV
	Phonological encoding	Mispronounced or ‘slurred’ speech, jargon, binary reversals, e.g. ‘Yes/No’	Reading aloud or writing non-words, e.g. proper names	Graded Non-word Reading Test^g^	NFV,LV
Delivery	Speech motor programming and articulation	Slow, hesitant, effortful speech, mispronounced or ‘slurred’ speech, monotonous, altered accent or singing	Production of syllable strings, e.g. ‘puh-kuh-tuh’	Apraxia Battery for Adults^h^	NFV,PD, PPAOS
Message understanding					
Perception	Decoding speech sounds	Better understanding of written vs spoken messages; ‘deaf’ behaviour	Compare understanding of spoken vs written commands	PALPA-3 ‘minimal pairs’ (phoneme) discrimination^i^	PWD
Meaning	Decoding grammatical relations	Confusion following more complex instructions	Difficulty following commands involving syntactic relations	PALPA-55 sentence comprehension^i^	NFV, LV
	Association with stored vocabulary	Asking the meaning of previously familiar words, using less precise or context-inappropriate terms, impoverished spoken and written vocabulary	Identifying items named by examiner, indicating meaning of words spoken by examiner, reading aloud or spelling irregular words, e.g. ‘sew’^b^	British Picture Vocabulary Scale^j^, Synonyms comprehension^k^	SV
Message repetition					
Words and phrases	Verbal working memory^c^	Usually no specific history; may have difficulty remembering new PIN or telephone numbers	Repetition of single words (effect of syllable number)	Polysyllabic word repetition^l^	NFV
			Repetition of phrases and sentences (effect of length)	WMS auditory digit span^m^	LV

The Table presents a framework for assessing speech and language functions on history and at the bedside. For each function, we indicate the primary progressive aphasia syndromes that characteristically affect that function (see Table 1) and examples of tests that might be used by a neuropsychologist to quantify the deficit; these are however not exhaustive and a number of additional tests are in widespread use, tailored to the disease stage and level of deficit

*DA* dynamic aphasia, *LV* logopenic variant primary progressive aphasia, *NFV* nonfluent variant primary progressive aphasia, *PA* progressive pure anomia, *PD* progressive dysprosodia, *PPAOS* primary progressive apraxia of speech, *PWD* progressive ‘pure’ word deafness, *SV* semantic variant primary progressive aphasia, *SYND* major syndromic associations

^a^Difficulty not obviously accounted for by degree of speech or vocabulary disintegration

^b^Surface dyslexia/dysgraphia’ (sounding out or spelling irregular words according to surface phonology) is a hallmark of semantic variant primary progressive aphasia

^c^Speech repetition tasks engage a number of component functions including accurate speech perception and motor output programing, however their primary purpose is to assess verbal working memory

^d^Gladsjo et al. (1992**)** *Assessment* 6: 147–178 (this test relies on an intact vocabulary as well as verbal generation per se, so should be interpreted in the context of verbal semantic competence, e.g. the British Picture Vocabulary Scale)

^e^McKenna and Warrington (1983) Graded Naming Test Manual, NFER-Nelson: Windsor

^f^Thompson (2011) Northwestern Assessment of Verbs and Sentences, Northwestern: Evanston

^g^Snowling et al. (1996) Graded nonword reading test, Thames Valley Test: Bury St. Edmunds

^h^Dabul (2000) Apraxia battery for adults. Second, Pro-Ed: Austin

^i^Kay et al. (1992) Psycholinguistic Assessments of Language Processing in Aphasia (PALPA), Psychology Press: Hove

^j^Dunn et al. (1982) The British Picture Vocabulary Scale, NFER-Nelson: Windsor

^k^Warrington et al. (1998) Neuropsychological Rehabilitation 8: 143–154

^l^McCarthy and Warrington (1984) Brain 107: 463–485 (this test in itself is an index of speech production but also provides a reference for interpreting verbal working memory effects on phrase repetition)

^m^Wechsler (1987) WMS-R: Wechsler Memory Scale-Revised Manual, Harcourt Brace Jovanovich: San Antonio (in the context of severe speech apraxia, this test can be modified to allow nonverbal responses such as pointing to numbers in an array, to avoid the confounding effects of motor speech impairment)

**Table 3:** Examples of spoken and written language output in patients with canonical syndromes of primary progressive aphasia

Syndrome	Speech transcriptions	Written sentences		
nfvPPA: prominent speech apraxia	The lady is drying a the plate, which she has washed. Meanwhile, she’s forgot… forgot to plug the s… silk. She’s forgot… She’s forgotten to pus put the plug in and she’s forgotten to turn off the water. And the boy is precariously standing on a stool to reach the cookies and he’s passing one cookie to his sister. And and the cur… curtains are quite open, revealing the their garden and a sort of shed	–	–	–
	[10 years]	–	–	–
nfvPPA: prominent agrammatism	Laughs} Obviously um there…. The man a boy um a cookie is on there. Um a daughter a no no um sister… And when she gonna throwen on them. And they broken and book on there on there stool on round by them. And lady and she was having a washing on there and they be taps on them and drain is come on the floor one there. Horrible {Laughs}A dire one there. And um…And this man are there bear maybe going in the went on there… working out the window. Maybe this little… little girl looking so on there	I have do pushed the door	My wish a happy Christmas to everyone	Dog walking muppett
	[5 years]	[2 years]	[4 years]	[4 years]
svPPA	Well there is a woman to her children, and it’s a house I suppose, and there’s a little window from there and I don’t quite know what that is going down, precisely, and then the children are… Cookie jar, I don’t understand that quite either what they’re doing. And gosh, that one’s foot is about to come off, oh dear {Laughs} Well those are people all down the end I suppose just that way, or maybe not, maybe it’s buildings, maybe it’s trees etc., I don’t know	I am having a stuppid remember	Now is the tyme for all good folcs to come to the aid of our party	I am sorry that I have no brain now
	[11 years]	[4 years]	[4 years]	[9 years]
lvPPA	Um… I see a mum washing, drying some um… {long pause} … plates. Crikey. With water running down. I see a young boy um in the… the other part of the um… {long pause} Trying, getting off or nearly getting off the… {long pause} … stool, with his… sister below	I caught a good crap when I was fishing	The keeper caught a difficult …	The cat sent on the …
	[4 years]	[2 years]	[4 years]	[8 years]

These language samples were all derived from different individuals. The speech transcripts represent attempts to describe the Cookie Theft scene from the Boston Diagnostic Aphasia Examination (Goodglass and Kaplan 1983), available at: http://images.slideplayer.com/14/4364218/slides/slide_23.jpg (digital wavefiles of the speech samples are provided separately with Supplementary Material on-line) The writing samples represent spontaneous responses when the patient was asked to produce a complete sentence de novo. For each case, the length of the patient’s history when the sample was collected is recorded below, demonstrating variable severity for a given illness duration across syndromes. The patient with prominent speech apraxia illustrates impaired delivery of the spoken message, with frequent stuttering sound duplications, ‘groping’ to reach the target sound and mis-articulated syllables; strictly there is no written analogue to speech apraxia per se (since this is a disorder of articulatory programming), though patients with nfvPPA commonly make phonological errors when writing due to an associated impairment of message assembly (see Table 2). The patients representing other syndromes here illustrate analogous deficits of spoken and written language output. The agrammatic nfvPPA cases show impaired structuring of verbal messages, with disordered sentence syntax and verb morphology and poverty of function words. The svPPA cases illustrate impoverished message content, with a relative dearth of specific, lower frequency vocabulary (particularly nouns), circumlocutions and surface dysgraphic errors (underlined), despite intact verbal structure (syllables and sentences). The lvPPA cases show marked word retrieval difficulty (impaired message content) reflected in prolonged pauses when speaking and trailing off of written sentences and in addition, incorrect syllable selection (impaired message structure; underlined) despite intact sentence construction; the speech sample of the lvPPA case here illustrates the challenge of reliably assessing spoken grammar in the setting of severe word-finding difficulty

*lvPPA* logopenic variant primary progressive aphasia, *nfvPPA* nonfluent–agrammatic variant primary progressive aphasia, *svPPA* semantic variant primary progressive aphasia

**Table 4:** ‘Clinical pearls’ in the diagnosis of progressive aphasia syndromes

Syndrome	Clinical observations
nfvPPA	Re-emergence of a childhood stammer may herald speech decline
	‘Binary reversals’ in conversation often occur early, and may extend to writing and nonverbal gestures: when required to select between alternatives (e.g., ‘yes/no’, ‘he/she’), the patient regularly produces the wrong response and will often spontaneously correct this [105]
	Late in the course, speech may become replaced by frequent laughter-like (‘gelastic’) vocalisations, unlike normal mirth or pathological affect [106]
	Naming and single word (particularly verb) comprehension deficits often develop [11, 107]
	Deficits of complex auditory processing may impair understanding of environmental sounds, emotional and other vocal signals (especially unfamiliar accents) [108–110], exacerbated in noisy environments or over the telephone
svPPA	Verbal knowledge deficits may appear first in more specialised lexicons previously mastered by that individual (e.g., flowers for a gardener; Greek playwrights for a classicist)
	In conversation, patients do not search for ‘lost’ words but often seem querulous and perplexed by vocabulary they encounter (in other PPA syndromes, patients tend to strive actively to find the word they need, with variable success); many compile personal ‘dictionaries’ to record the meanings of words they no longer understand
	Auditory symptoms are prevalent (including tinnitus, hyperacusis, aversion to particular environmental noises), not adequately explained by peripheral hearing impairment and likely central in origin [59, 111]; families may interpret patients’ difficulty understanding others as ‘deafness’
	Numerical and geographical references (times, dates, distances, quantities, locales) may ‘scaffold’ the patient’s conversation (Supplementary sound file 3); these more abstract, autonomous domains may (like music) be oases of relative semantic competence [112–114]
lvPPA	Verbal working memory impairment may be brought out by a series of sentence repetitions: phonological errors appear and the target sentence becomes a truncated and inaccurate replica (due to progressive overloading of the exhausted verbal buffer)
	During sentence repetition tasks, there may be repeated attempts to approach the target via a series of substitutions and approximations, resembling ‘conduite d’approche’ in conduction aphasia [115]
	Jargon and neologisms may occur in conversation or naming tasks (e.g., ‘dajent’ for kangaroo, ‘fishgii’ for buoy); rare in other neurodegenerative syndromes [116, 117]
	There may be prominent verbal semantic deficits (possibly indicating separate sub-syndromes [73])

This Table presents some clinical observations that are not currently emphasised in standard diagnostic formulations but which we have found useful in the bedside diagnosis of the major syndromes of primary progressive aphasia

*lvPPA* logopenic variant primary progressive aphasia, *nfvPPA* nonfluent–agrammatic variant primary progressive aphasia, *svPPA* semantic variant primary progressive aphasia

## A clinical framework for assessing language functions in primary progressive aphasia

When confronted by an aphasic patient, it is important firstly to establish the context of the language disturbance. This usually requires the help of an informant who knows the patient well and can supply reliable background information. A diagnosis of PPA requires that speech or language dysfunction was the initial and most salient clinical complaint (see Table S1). However, the patient’s previous verbal skills (including formal education, occupation, bilingualism or any specific developmental difficulties such as stammering or dyslexia) are relevant to interpreting current deficits. It is also necessary to determine the extent of any uncorrected peripheral hearing or visual impairments as these can impact significantly on everyday communication and performance on language tests. In defining the history of the language problem, it is essential to establish the circumstances of onset and very first symptoms (often noticed by the patient’s family), overall duration and tempo. The length of the history bears strongly on the interpretation of deficits, since PPA syndromes tend to converge over time [17]. In PPA, a history of gradual, but unrelenting decline over a number of months or several years is typical, but some apparent fluctuation is not uncommon, particularly under conditions that stress the language system, such as public speaking or conversations by telephone or in a non-native tongue. There may have been a sentinel event such as a family celebration or minor head injury that first drew attention to the patient’s difficulties; informants may interpret this as an acute onset but a searching history usually reveals a more insidious prodrome.

The profile of the patient’s language dysfunction then allows the clinico-anatomical syndrome to be characterised (see Table 1). Fundamentally, language supports communication—the understanding, creation and delivery of messages. In assessing a patient’s speech, it is useful to analyse the various stages at which the idea for a message is first generated, the content (or vocabulary) of the message, its structure (assembly) and delivery. Similarly, in assessing understanding of language, it is useful to analyse the separable stages at which messages are perceived and then invested with meaning. These operations are differentially vulnerable to particular PPA syndromes and can be explored using targeted questions on history and a small set of core language tests (Table 2). The patient’s use of written language typically echoes the speech disorder as the illness evolves. Examples of patients’ transcribed spoken and written productions are presented in Table 3 (corresponding speech sound files are provided in Supplementary Material on-line).

In neurology, the history generally suggests the diagnosis while the examination corroborates the historical suspicion. This precept is equally valid for language disorders, with the caveat that certain aspects of language are difficult to differentiate on the story alone. One key example (not often called upon in everyday communication) is the ability to repeat messages verbatim, which is central to the characterisation of PPA (see Table 1) and should be examined explicitly. Like the testing of pupillary and spinal reflexes in general neurology, certain language tests such as speech repetition or picture naming rapidly assay a number of connected neural operations: if such tests are performed normally, this demonstrates the overall integrity of the system but if a problem is found, it is necessary to establish where in the system it lies. The most important principle in examining speech is to obtain an adequate sample; for this purpose, it is convenient to carry a picture that will encourage the patient to talk and provide a prop for directed tests (one example is shown in Fig. 2).

Alongside the core clinical tests in Table 2 we list some more formal equivalents that might be administered by a neuropsychologist. However, neuropsychological assessment does not simply endorse the bedside impression. If available, it adds considerable value, particularly in quantifying language capacities in relation to standardised population norms and in the context of estimated premorbid ability, in tracking change in language functions over time and in measuring associated capacities that together with aphasia define the overall cognitive phenotype and may also affect the assessment of language.

## Canonical syndromes of primary progressive aphasia: nonfluent–agrammatic

### Clinical presentation

Patients with nfvPPA present with slow, effortful, hesitant and distorted speech (Table 3; Supplementary sound files 1 and 2). Speech sound errors are generally prominent and there is often a history of ‘slurring’ or mispronunciations. Words tend to be missed out and conversation is sometimes strikingly telegraphic; errors of grammar (mainly affecting syntax, function words such as articles and conjunctions and verb usage) typically emerge and sometimes dominate the presentation [11, 18]. Inability to understand more complex conversations or instructions may signify impaired comprehension of sentences, which is generally integral to any grammatical deficit [19]. Speech is usually very much more affected than written communication at the outset and patients tend to resort increasingly to nonverbal means of expression, manifestly frustrated by their inability to communicate.

On examination, there is usually marked difficulty producing polysyllabic words and sequences of syllables (e.g., ‘puh-tuh-kuh’) to command, due to impaired motor programming of speech and reduced articulatory agility. This can be brought out by asking the patient to repeat longer words or read aloud. The listener is left with an almost painful sense of the patient’s struggle to speak (not experienced with other forms of PPA). In contrast to peripheral dysarthrias which tend to provoke stumbling consistently over particular sounds, the misshapen speech of patients with nfvPPA is protean, with characteristic ‘groping’ after the target sound: ‘speech apraxia’ [20]. This is often accompanied by apraxia of posed orofacial movements such as yawning or whistling, disproportionate to any limb apraxia [21]; asked to perform an orofacial gesture, the patient may emphatically echo the command (‘Cough!’) while remaining quite unable to enact it. Speech sound errors can be classified according to whether syllables are wrongly selected (‘phonemic’ or ‘phonological’ errors) or misformed during execution (‘phonetic’ or articulatory errors). These arise at different stages during message production but often defy explicit categorisation in the clinic and the distinction is seldom of practical importance. It is useful to examine a specimen of the patient’s writing (Table 3): besides revealing spelling (phonological dysgraphic) errors, this is a more reliable index of associated agrammatism than the patient’s speech, which may be constricted by the sheer effort involved.

The clinical spectrum of nfvPPA is the most diverse of the canonical PPA syndromes, with a number of variant sub-syndromes (see Table 1). The most important of these is ‘pure’ progressive speech apraxia associated with orofacial apraxia, but without agrammatism or other aphasic features, which has been proposed to constitute a distinct entity [22, 23]. While apraxia of speech may indeed be relatively pure at presentation [11], in our experience most of these patients do in time develop aphasia, initially detected on detailed neuropsychological assessment. Some clinical ‘pearls’ we have found useful in the diagnosis of nfvPPA are presented in Table 4.

### Neuroanatomy

This syndrome is associated with atrophy of inferior frontal gyrus (‘Broca’s area’) and insula cortex in the dominant hemisphere (Fig. 1), with variable extension along and around the superior temporal gyrus. These brain regions play fundamental roles in language output, motor speech programming and sentence processing [10]. Atrophy is generally best appreciated as widening of the left Sylvian fissure on a T1-weighted coronal MRI scan [24]. However, this may be subtle on cross-sectional imaging and is easily overlooked on ‘routine’ reporting lists, by even experienced observers [25]. Moreover, rotated slices may simulate asymmetry; scrolling through a number of slices is useful to check that the direction of any apparent asymmetry is consistent (and therefore real). A neuroradiological phenotype of homologous right-sided peri-Sylvian atrophy is recognised, though its clinical correlates remain ill-defined [26, 27]; several of our patients with this finding have had notable central nonverbal auditory deficits or dysprosody [28].

### Key associations

General intellect is often remarkably well preserved, though a degree of executive dysfunction is usual and may be accompanied behaviourally by apathy or impulsivity [29, 30]. Depression can be significant, particularly as insight is usually retained. Many patients with nfvPPA will develop Parkinsonism, often evolving into a progressive supranuclear palsy or corticobasal syndrome with associated supranuclear gaze palsy, postural instability, pseudobulbar dysfunction and limb apraxia, dystonia or ‘alien’ phenomena [31, 32].

The pathological associations of nfvPPA are (in keeping with the clinical spectrum) more heterogeneous than other PPA syndromes. A majority of patients will have a tauopathy such as progressive supranuclear palsy or corticobasal degeneration at post-mortem though a substantial (and still uncertain) minority represent TDP-43 or Alzheimer pathology [3, 12, 33–35]. While there are currently few reliable predictors of underlying pathology in individual patients [36], prominent apraxia of speech and parkinsonism are more closely associated with tauopathy than with TDP-43 pathology [12, 35]. nfvPPA is less likely to be genetically mediated than the behavioural variant of frontotemporal dementia though it is somewhat more heritable than other PPA syndromes, around 30% of patients having a relevant family history [37]. Causative mutations in all major (*GRN, MAPT, C9orf72*) genes causing frontotemporal dementia have been identified and at least some of these genetic forms may prove clinically distinct with more detailed phenotyping [11].

## Canonical syndromes of primary progressive aphasia: semantic

### Clinical presentation

In striking contrast to nfvPPA, patients with svPPA exhibit well structured, well articulated language that is relentlessly bereft of substance (Table 3; Supplementary sound file 3). This typically begins as difficulty finding words (particularly nouns)—sometimes described as losing ‘memory for names’—and an inability to express thoughts with precision. The patient’s verbal messages become progressively more circumlocutory and empty, as fine-grained content (less frequently used vocabulary, such as ‘dachshund’ or ‘ladybird’) is replaced by increasingly generic ciphers (‘animal’, ‘thing’). Blunting of verbal nuance in svPPA may predate diagnosis by many years [38]. The true nature of the deficit is revealed in a history (almost pathognomonic for svPPA) of asking the meaning of previously familiar words (‘What’s broccoli?’): this is not merely a problem of accessing words in memory, but erosion of vocabulary itself. Indeed, svPPA is the paradigmatic disorder of semantic memory, the cognitive system that stores (rather than the autobiographical events that populate ‘episodic’ memory) knowledge about objects and concepts and allows us to attribute meaning to the world at large [6, 39]. The language deficit in svPPA is fundamentally associated with loss of meaning about objects and people. While language impairment usually leads the presentation, deficits of nonverbal knowledge inevitably appear later in the course and ultimately blight all sensory channels [39–42]. More rarely, patients present with inability to recognise objects (visual agnosia) or familiar people (prosopagnosia) by sight.

Earlier in the course of the illness, the conversation of patients with svPPA is easily passed as normal by the casual listener, due to its well preserved surface structure and fluency, even garrulousness [11]. However, closer attention generally reveals severe anomia. Because anomia is a common feature of a number of aphasias, it is important to distinguish carefully those cases (for example, in svPPA) where this follows degradation of the word store (primary semantic impairment) from the more usual scenario, in which retrieval of words from storage is principally affected. It is failure to comprehend or recognise words and objects rather than anomia per se that defines a semantic deficit. Impaired comprehension of single words in svPPA can be demonstrated by asking the patient to describe an item nominated by the examiner or to select it from an array or scene (see Fig. 2).

Assessment of other language channels corroborates the semantic deficit. When reading aloud or writing, patients with svPPA characteristically ‘regularise’ words according to superficial phonological ‘rules’ in place of learned vocabulary (e.g., sounding ‘island’ as ‘izland’ or ‘sew’ as ‘soo’): so called ‘surface’ dyslexia or dysgraphia (Table 3). English is a particularly fertile field for such deficits as it is replete with irregular ‘exception’ words, but analogous examples exist in other languages (disproportionately affecting, for example, kanji versus kana script in Japanese [43]). Assessment of nonverbal semantic domains generally requires more detailed neuropsychological assessment, though in clinic visual knowledge might be conveniently sampled (within the limits of verbal comprehension and without requiring naming) by asking the patient to indicate the purpose of a familiar tool (such as a comb or stapler), to identify associations of a pictured item (‘which thing could be used in the garden?’; Fig. 2) or to supply biographical information from photographs of familiar people. Across verbal and nonverbal semantic domains, loss of meaning in svPPA follows a stereotyped pattern. More specific knowledge about less familiar (low frequency) and atypical items is lost before knowledge of highly familiar and typical items; failures of recognition are accompanied by ‘over-generalisation’ errors that tend to regularise objects to a generic type (for example, the patient may draw a four-legged peacock or a rhino lacking its horn); and errors are highly consistent over time, so that the meanings of words and objects, once lost, are irretrievable [44, 45]. These features of svPPA have informed neural computational models of the underlying cognitive architecture of semantic memory and its breakdown [46, 47]. Some clinical ‘pearls’ relevant to svPPA are listed in Table 4.

### Neuroanatomy

On neuroimaging, svPPA has a hallmark pattern of asymmetric, focal cerebral atrophy chiefly involving the dominant anteroinferior and mesial temporal lobe, including amygdala and anterior hippocampus [9, 48]. This is most easily visualised on a T1-weighted coronal MRI scan (Fig. 1). The profile of atrophy shows a clear gradient within the temporal lobe, with ‘knife-blade’ destruction of the pole and relatively sparing of superior temporal gyrus and more posterior temporal cortices. This signature is consistently observed across patients and unmistakeable; in our experience, it is invariably present at diagnosis in typical svPPA and indeed (in contrast to nfvPPA) often ‘the scan is worse than the patient’. Over time, atrophy spreads to involve more posterior temporal regions and homologous gyri in the contralateral temporal lobe as well as orbitofrontal cortex [49, 50]: regions that together constitute the core of the brain’s semantic memory network [39, 47]. This distinctive atrophy profile has provided an important neuroanatomical grounding for cognitive models of svPPA, according to which anterior temporal cross-modal ‘hub’ cortex interacts with more posterior, relatively modality-specific cortices across both left and right temporal lobes [47].

Some patients exhibit a ‘mirror’ profile of predominant right anterior temporal lobe atrophy. For unknown reasons, these cases are rarer than their leftward-asymmetric counterparts and usually present with profound disturbances of social and emotional behaviour or prosopagnosia, indicating the breakdown of knowledge about people [51, 52].

### Key associations

A behavioural syndrome similar to that defining the behavioural variant of frontotemporal dementia characteristically develops in svPPA and indeed, these syndromes can occasionally be difficult to distinguish, even after a careful history. Initially, behavioural features in svPPA may be quite subtle, but tend to manifest earlier and more floridly in patients with more marked right (non-dominant) temporal lobe involvement and become universal as the march of disease involves the frontotemporal networks that regulate social responsiveness [39, 47]. Symptoms such as absent or misplaced empathy, social disinhibition and faux pas, a more fatuous sense of humour and pathological sweet tooth are common in both svPPA and behavioural variant frontotemporal dementia [29, 53–57]. Within this spectrum, certain behavioural features, such as food faddism, exaggerated reactions to pain and ambient temperature, behavioural rigidity with clock-watching and obsessional interest in numbers, puzzles (especially Sudoku and jigsaws) and music (‘musicophilia’) seem particularly linked to svPPA [30, 53, 58–60]. A unifying theme here may be impaired understanding of emotional and somatic signals due to both deficient and over-generalised responses to sensory information [41, 42, 55, 56, 61], analogous to recognition failures and ‘regularisation errors’ in other cognitive domains. An impoverished concept of self due to diminished awareness of bodily signals may contribute to reduced empathy and an increased rate of suicidality in svPPA relative to other neurodegenerative syndromes [61, 62]. Insight and awareness of deficits often appear to be retained, but may be superficial or incomplete. In contrast to nfvPPA, associated neurological signs are not typically found in svPPA, though parkinsonian or motor neuron features may develop later in the course [31, 63].

Completing the picture of a highly coherent clinical, anatomical and pathological syndrome, most cases of svPPA have TDP-43 (type C) pathology at post-mortem [12, 33, 35]. Primary tauopathies and Alzheimer’s disease account for a small minority and may have certain distinguishing phenotypic markers (for example, prominent acalculia or extrapyramidal signs in association with Pick’s disease pathology [12, 35]). Most cases are sporadic though occasional pathogenic mutations are reported [4] and may be relatively more likely if motor features are present (e.g., associated motor neuron disease with *TBK1* mutations [64]).

## Canonical syndromes of primary progressive aphasia: logopenic

### Clinical presentation

The most recently described of the major PPA syndromes is (in more than one sense) the most problematic. This is due chiefly to the issue of demarcating it clinically from typical Alzheimer’s disease and the lack of a readily agreed, unifying syndromic deficit in lvPPA to set against the speech production (message production) failure that defines nfvPPA and the word comprehension (message understanding) failure that defines svPPA. The clinical picture in lvPPA is usually dominated by word-finding difficulty and conversational lapses, for which the syndrome is named (Greek, ‘lack of words’; Table 3, Supplementary sound file 4). Early on, ‘tip-of-the-tongue’ hesitations are often prominent. Some patients develop a rather mannered style of conversation, likened by one spouse to a ‘Jane Austen character’. Interrupted sentences that tend to trail off may give the impression of agrammatism though without the frank syntactic dislocations of nfvPPA [18]. Speech sound or spelling errors are frequently described. The patient may struggle to understand more complex sentences and to hold verbal information in mind. While language is (by definition) the leading and dominant issue, there is frequently a history of associated extra-linguistic difficulties extending to the realms of memory (e.g., forgetfulness, repetitiveness or route-finding problems), praxis (e.g., use of work equipment, tools or household gadgets) or visuo-spatial awareness (e.g., inability to judge distances accurately, find exits or locate items in plain sight) [65, 66].

On examination, the patient’s speech is derailed by word retrieval pauses and (often marked) anomia, though this is usually not as severe (from such an early stage) as in svPPA. This syndrome illustrates the difficulty of dichotomising PPA syndromes as ‘nonfluent’ versus ‘fluent’: patients with lvPPA in general do not talk fluently, yet their speech sounds quite different to the mutilated utterances of nfvPPA (compare Supplementary sound files 1, 2 and 4). Generally, phonological speech sound errors can be detected, usually taking the form of syllable misselections that are enunciated clearly and not accompanied by the false starts and distortions that characterise nfvPPA. Reading aloud is similarly marred by syllabic substitutions, highlighted by sounding out non-words (e.g., proper names) that rely on phonological decoding rather than learned vocabulary. Analogous errors of written spelling are often evident (Table 3). The diagnostic feature of lvPPA that distinguishes it from other PPA syndromes is early and disproportionate difficulty repeating heard phrases and sentences versus single words [67] (see Table 2). This signifies an impairment of verbal (phonological) working memory, also indexed as a reduced auditory span for repetition of random digit strings [16]. While digit span is often also reduced in nfvPPA, in that syndrome repetition of single words and repetition of phrases are comparably degraded. Other dominant (and often, bi-hemispheric) posterior cortical signs such as limb apraxia and visual apperceptive agnosia can frequently be elicited in lvPPA. This extra-linguistic cognitive phenotype tends to be more extensive and severe than in other PPA syndromes at a comparable stage of clinical evolution (see Fig. 1). Some clinical ‘pearls’ relevant to lvPPA are presented in Table 4.

### Neuroanatomy

The key neuroimaging association is asymmetric atrophy mainly involving the temporo-parietal junction zone of the dominant hemisphere, appearing as widening of the posterior left Sylvian fissure on a T1-weighted coronal MRI scan [68] (Fig. 1). This locus potentially accounts for many features of the language phenotype, since posterior superior temporal and inferior parietal cortices are intimately involved in decoding speech sounds and activating phonological representations to link verbal semantic stores to language output [10, 16, 69]. However, there is often extension of atrophy more anteriorly with involvement of other structures (notably the hippocampi) and the overall extent and pattern of atrophy varies widely between individual patients.

### Key associations

A number of patients with lvPPA exhibit generalised anxiety, irritability and increased clinging (emotionally and physically) to their primary caregivers [29, 70, 71]. Similar behavioural features may occur with posterior cortical atrophy (the ‘visual’ Alzheimer variant syndrome) or indeed in typical Alzheimer’s disease. Neurological signs are usually sparse [32], but include myoclonus, which may be peri-oral. Some patients go on to develop a corticobasal syndrome [71].

In most cases, lvPPA is a variant presentation of Alzheimer’s disease. This explains the extensive phenotypic overlap of lvPPA with both later-stage typical Alzheimer’s disease and the posterior cortical atrophy variant, corroborated at post-mortem as well as on neuropsychological, CSF (raised phosphorylated tau levels, raised ratio of total tau to beta-amyloid_1-42_) and amyloid-PET case series [35, 70–72]. On the other hand, cases of lvPPA lacking Alzheimer markers are consistently represented across series; the pathological substrates have not been clarified, but these may point to separable clinico-anatomical sub-syndromes [16, 68, 71–73]. Although lvPPA is generally a sporadic disorder, some caution is called for in cases without Alzheimer markers, since pathogenic progranulin gene mutations have been reported in this subgroup [11, 68].

## Toward the diagnosis: a path with pitfalls

Accurate and early identification of PPA syndromes is essential for clinical counselling and planning appropriate management. Beyond clinical characterisation, molecular stratification will become increasingly important with trials of candidate disease-modifying treatments on the horizon [35]. However, most patients presenting with a language complaint will not have PPA and further, a number of patients with PPA (as many as 40% in some series) do not conform closely to one of the canonical syndromic diagnoses [3, 13, 15, 16, 34]. Some of these less common, atypical variants are in Table 1.

In Fig. 3, we present a ‘roadmap’ for diagnosis of PPA syndromes that we have found useful in clinic. Key diagnostic decision ‘forks’ rest on the historical and examination findings outlined in Tables 1 and 2 and on neuropsychological assessment where available. Structural brain imaging (ideally, MRI) is essential in all cases of suspected PPA, both to rule out other causes of progressive language failure and more positively, to identify features of particular radiological phenotypes (Fig. 1). Where initial MRI features are borderline (notably in nfvPPA and lvPPA) it may be helpful to repeat the scan after an interval of a year or so, as directly comparing serial studies of the culprit brain region is often revealing. Functional MRI and FDG-PET in PPA are not in routine clinical use though there is considerable potential interest in applying such techniques to define aberrant and compensatory language network changes, with a view to future therapeutic trials [74, 75].

Having arrived at a syndromic diagnosis, it may be appropriate to investigate further to determine the underlying proteinopathy—including CSF examination or amyloid-PET scanning for Alzheimer neurodegeneration markers. These ancillary investigations may be relevant, for example, in deciding to trial a cholinesterase inhibitor in a patient with nfvPPA or more generally, in forecasting the overall outlook of the illness in earlier stage disease. We discuss the possibility of genetic risk in all younger patients with nfvPPA and genetic screening should be considered in any patient with PPA who has a relevant family history, particularly where there this raises suspicion of a frontotemporal dementia. Discussions in clinic around genetic testing broach a number of sensitive issues and should ideally involve other family members.

We next consider some common potential pitfalls on the path to diagnosis.

### The patient with late stage or ‘global’ aphasia

All PPA syndromes tend ultimately to give rise to global language failure with mutism or sparse, stereotyped utterances [76] as well as more widespread cognitive decline. Accordingly, diagnosis later in the course may be more informed by associated neurological features such as Parkinsonism. On the other hand, ‘mixed’ aphasia does not of itself signify advanced disease: some patients exhibit deficits that transcend canonical syndromic boundaries early on (see Table 1). In our experience, this includes cases with progranulin mutations [11] and Alzheimer’s, Pick’s and other pathological associations are reported [16, 35].

### The unhelpful scan

Neuroimaging findings in nfvPPA and lvPPA can be subtle and an equivocal scan does not rule out the diagnosis. A related issue concerns the diagnostic relevance of visualised abnormalities; PPA syndromes can be caused by unusual pathologies (for example, primary leukodystrophies and prion disease), but most meningiomas and arachnoid cysts will be incidental. Cerebrovascular changes of small vessel ischaemia are commonly found in older patients but seldom if ever cause a canonical PPA syndrome (though word-finding difficulty commonly accompanies vascular cognitive impairment).

### The older patient

It is likely that PPA is underdiagnosed in older patients in whom language deficits are more likely to be ascribed uncritically to Alzheimer’s disease or undifferentiated ‘dementia’ [4]. On the other hand, impaired word retrieval commonly occurs in amnestic Alzheimer’s disease, frontotemporal dementia and other diseases and may be a salient early feature even if it does not dominate the presentation [10, 77]. These patients often do poorly on naming tasks and may substitute semantically related ‘paraphasias’ (e.g., ‘rhino’ instead of ‘hippo’). The speech of advanced typical Alzheimer’s disease has characteristics similar to lvPPA, though close analysis suggests that these syndromes are linguistically distinct at earlier stages [78]. Accurate diagnosis is worthwhile, given the quite different management issues that these groups present.

### The patient with comorbidities

Interpretation of apparent language deficits should be cautious in older patients with a history of developmental dyslexia or longstanding peripheral hearing loss, especially when the recent symptoms target phonology or articulation and if additional cognitive involvement is subtle. Often a period of follow-up will establish the nature of the deficit. Performance on language tests should always be calibrated for premorbid literacy skills in the test language.

### The worried well or ‘functional’ patient

Word-finding difficulty is a very common complaint in patients attending memory clinics [10]. Many will be experiencing the effects of normal ageing and intercurrent stressors; they typically describe inefficiency in recalling names or clearly expressing their thoughts when preoccupied or fatigued and have no evidence of language deficits on objective testing. A taxonomy of neurologically unexplained, ‘functional’ or ‘non-organic’ speech disorders has been described [79]. In our experience, these cases are rare and tend to present as excessively deliberate, but immaculately executed speech or with isolated disturbances of prosody (‘melody’ of speech). Dysprosody is a regular accompaniment of nfvPPA and ‘pure’ primary progressive dysprosodia (leading to the development of other aphasic features) is a rare satellite syndrome in the PPA spectrum (Table 1). Indeed, ‘foreign accent syndrome’ has been described as a presentation of PPA [80]. Our patients with nfvPPA (and occasional cases of primary progressive dysprosodia) have exhibited degraded native accents or loss of a previously competent second language accent, rather than developing a facsimile foreign accent. ‘Organic’ dysprosody tends to be brought out by circumstances (such as singing or reciting) calling for heightened control of vocal intonation [26, 81].

When interpreting a language disorder as ‘neurologically unexplained’, it is important to appreciate that bona fide PPA syndromes can have quite counter-intuitive manifestations. An expert second opinion may be useful and the passage of time (and lack of clinical and radiological evolution) often clarifies the situation.

## An outline of management

Patients with PPA generally live for a number of years following diagnosis, with evolving deficits and specific needs at each stage of the illness. Widely accepted clinical staging markers are presently lacking, however, the major PPA syndromes collectively raise similar management challenges and these broadly require the integration of non-pharmacological and pharmacological approaches.

### Non-pharmacological strategies

Management begins with diagnosis; this is often delayed due to the lack of experience with these conditions in the wider medical community and the value of a clear explanation for patients and families should never be underestimated. Diagnosis supports future planning (including discussions around end-of-life care) and mobilisation of appropriate social services. Supportive care is still the mainspring of management for all PPA syndromes. Patients and caregivers need clear advice about driving safety, work arrangements, safeguarding and finances (particularly in younger patients who may have dependent children or elderly relatives). Later in the course, and especially in nfvPPA, dysphagia (due to motor dyscontrol and/or impulsivity) may become a significant issue necessitating expert advice from a dietician or speech therapist and where appropriate, consideration of assisted feeding. Patients should also be monitored for the emergence of other motor and neurological features that impact on mobility and activities of daily life. Early detection of physical deficits is key because these tend to herald a step change in functional status and care requirements. It may be useful to provide the patient and caregiver with a medical card or bracelet as the ability to communicate diagnosis and needs fails due to severe communication and/or behavioural decline. If available, involvement in a lay support group dedicated to PPA or frontotemporal dementia often provides much-needed psychological support and practical advice (in the United Kingdom, see: http://www.raredementiasupport.org/ppa/). Support and respite for caregivers are often overlooked, but vital to maintaining patients in the community.

Speech and language therapy in PPA has an important role in providing communication aids and strategies. Even simple measures such as picture books and cards listing frequent and important words and phrases that the patient can carry may be of great practical value (particularly in nfvPPA). More structured therapy aims to provide person-centred and goal directed interventions to alleviate impairment (e.g., word-relearning tasks) and to maintain daily life functioning (e.g., ordering in a coffee shop) [82, 83]. These can in principle be tailored to the particular PPA syndrome: for example, word-relearning techniques might focus on object features (use, location, appearance) in patients with svPPA or phonology (rhyming, first and last sound identification) in lvPPA [84], while orthographic cues to word production can be targeted in nfvPPA [85]. In practice, however, combined approaches have often been used [84]. Communication skills training is informed by experience in stroke aphasia and aims to enhance strategies that facilitate communication (e.g., gesture) while avoiding communication barriers (e.g., interruptions, abrupt topic changes). Augmentative and alternative communication devices may help patients with nfvPPA and limited verbal output, but preserved comprehension [86]. Adapting everyday technology such as smartphones and total communication strategies incorporating photos and pictures may enable continuation of daily activities such as shopping or cooking [87, 88]. Unfortunately, gains from non-pharmacological therapies are usually modest and there is little evidence for generalisation of effects or lasting benefit in daily life [83, 84]. It is important that new approaches continue to be developed and are assessed in adequately powered and controlled studies, with a view to a future where cognitive rehabilitation may be deployed in conjunction with disease modifying pharmacotherapy.

### Pharmacological interventions

There are currently no disease modifying treatments for PPA and evidence for efficacy of symptomatic treatments is scant. We have a low threshold for trying a cholinesterase inhibitor or memantine in patients with lvPPA and nfvPPA (where Alzheimer’s pathology is a consideration), though any benefit is usually modest and care is needed to avoid exacerbating behavioural symptoms [89, 90]. Memantine appears to be well-tolerated in svPPA and nfvPPA, but clear evidence of benefit is lacking [91]. Selective serotonin reuptake inhibitors should be considered in patients with comorbid depression or anxiety and may help to settle behavioural symptoms such as impulsivity and aggression, particularly in svPPA [92]. Newer generation neuroleptics may be indicated to manage severe agitation or psychotic symptoms later in the illness.

## Conclusions and future directions

Recognition of the major PPA syndromes has transformed our understanding of the language system and given us a new picture of selective neural vulnerability in degenerative brain disease. However, this dramatic progress should not obscure the many remaining difficulties surrounding these conditions. Ultimately, effective treatment of PPA will depend on both earlier and more accurate diagnosis (improved syndrome characterisation), more accurate disease and disability staging and identification of new biomarkers that can target tissue pathology and track therapeutic effects dynamically, in advance of irrecoverable brain atrophy (improved signalling of underlying proteinopathy [93]). A successful PPA biomarker will need to encompass substantial individual variation within syndromic categories (see, e.g., Table 3).

To realise these ambitions may require a reformulation of the ‘language-led dementias’ in more fundamental, pathophysiological terms. Emerging evidence suggests that generic disorders of nonverbal auditory information processing may underpin the canonical PPA syndromes [40, 94–96] and that these syndromes may further be stratified by profiles of autonomic reactivity to emotional and other salient stimuli [61, 97, 98]. This evidence dovetails with the recent finding that implicit auditory sequence learning is retained in nfvPPA [99]. Such observations could motivate novel biomarkers and treatment strategies that do not depend on specific language capacities (a practical advantage in mounting large-scale, international trials in PPA). There is currently considerable interest in the application of molecular ligand neuroimaging techniques (including new tracers that can demonstrate tissue deposition of pathogenic proteins other than beta-amyloid) in PPA and other dementias [100]. Such techniques promise to delineate proteinopathies in vivo more reliably than is currently feasible; multivariate approaches combining ligand imaging with pathophysiological indices may constitute a powerful and dynamic signal of the underlying disease. Finally, more work is needed to establish rational retraining and functional rehabilitation interventions in PPA: in future, such interventions may amplify disease modifying therapies and bridge the gap from clinic to patients’ wider lives.

## Electronic supplementary material

Below is the link to the electronic supplementary material.

Supplementary material 1 (MP3 3616 kb)

Supplementary material 2 (MP3 2506 kb)

Supplementary material 3 (MP3 1086 kb)

Supplementary material 4 (MP3 1636 kb)

Supplementary material 5 (DOCX 14 kb)
