# Supplementary material for: Primary progressive aphasia: a clinical approach
Source: J Neurol. 2018 Feb 1;265(6):1474–90. doi: 10.1007/s00415-018-8762-6 (PMC5990560; doi:10.1007/s00415-018-8762-6)
Supplement: Supplementary file 5 — Supplementary material 5 (DOCX 14 kb) [file 415_2018_8762_MOESM5_ESM.docx]

**SUPPLEMENTARY MATERIAL. Primary progressive aphasia: a clinical approach,**

**by CR Marshall et al**

**Table S1.** Current consensus criteria for primary progressive aphasia (after Gorno-Tempini et al., *Neurology* 2011; **76**(11): 1006-14)

| Level diagnosis | **nfvPPA** | **svPPA** | **lvPPA** |
| --- | --- | --- | --- |
| **Clinical** | *At least one of:* | *Both of:* |  |
| *Core features* | Agrammatism in language production | Impaired confrontation naming | Impaired single-word retrieval in spontaneous speech and naming |
|  | Effortful, halting speech with inconsistent speech sound errors and distortions (speech apraxia) | Impaired single-word comprehension | Impaired repetition of sentences and phrases |
| *Other features* | *At least two of:* | *At least three of:* | *At least three of:* |
|  | Impaired comprehension of syntactically complex sentences | Impaired object knowledge, particularly for low-frequency or low-familiarity items | Speech (phonologic) errors in spontaneous speech and naming |
|  | Spared single-word comprehension | Surface dyslexia or dysgraphia | Spared single-word comprehension and object knowledge |
|  | Spared object knowledge | Spared repetition | Spared motor speech |
|  |  | Spared speech production (grammar and motor speech) | Absence of frank agrammatism |
| **Imaging-supported** | *At least one of:* | *At least one of:* | *At least one of:* |
|  | Predominant left posterior fronto-insular atrophy on MRI | Predominant anterior temporal lobe atrophy | Predominant left posterior peri-sylvian or parietal atrophy on MRI |
|  | Predominant left posterior fronto-insular hypoperfusion/metabolism on SPECT /PET | Predominant anterior temporal hypoperfusion/metabolism on SPECT /PET | Predominant left posterior peri-sylvian or parietal hypoperfusion/ metabolism on SPECT/PET |
| **Pathologically definite** | *At least one of:* | *At least one of:* | *At least one of:* |
|  | Histological evidence of specific neurodegenerative pathology | Histological evidence of specific neurodegenerative pathology | Histological evidence of specific neurodegenerative pathology |
|  | Known pathogenic mutation | Known pathogenic mutation | Known pathogenic mutation |

The current consensus proposes clinical, neuroimaging-supported and pathologically definite criteria for the clinical and research diagnosis of the major syndromes of primary progressive aphasia. An imaging supported or pathologically definite diagnosis rests on a clinical diagnosis of the relevant progressive aphasia syndrome. A clinical diagnosis of any of these syndromes rests on meeting all three of the following inclusion criteria: most prominent clinical feature is language decline, language deficits are the principal cause of impaired daily living, aphasia is the most prominent deficit at symptom onset. The diagnosis of primary progressive aphasia is *excluded* by any of the following: pattern of deficits is better accounted for by another neurological or medical disorder, pattern of deficits is better accounted for by a psychiatric diagnosis, prominent initial episodic memory or visuoperceptual deficits, prominent initial behavioural disturbance. lvPPA, logopenic variant primary progressive aphasia; nfvPPA, nonfluent-agrammatic variant primary progressive aphasia; svPPA, semantic variant primary progressive aphasia.
